# Supplementary material for: Chromothripsis during telomere crisis is independent of NHEJ, and consistent with a replicative origin
Source: Genome Res. 2019 May;29(5):737–49. doi: 10.1101/gr.240705.118 (PMC6499312; doi:10.1101/gr.240705.118)
Supplement: Supplemental Material [file supp_gr.240705.118_Supplemental_file_1.zip › contigs/annotated_contigs/DB79/contig.2.DB79_length_587_mean_cov_14.6030664395.docx]

**DB79_length_587_mean_cov_14.6030664395**

```
AATGTGGAAGTAAAAATTAAGAGTAAGCACTAAATCAGTAGAAACTTAATGTTTAGTTTTAAAATTAGCAGCAGAAAATAATGTATCAC
 >chr3:182271499-182271775 - E=2e-151
TTTAACAGAAAGTAGGAATGGGGAAAAAATGAAAGCGAAAGAAAAGAACAGTAAATAGAAAACACAAAATGAAATGGTAGAAATAAAGC

CGAATACATCAGTAATCATTAATTCTGGGAGGTTGAGGATGCAGTGAGCCATGATCATGCCATTGCACTCTAGCAGGGTAAGACCTTGT

ATATATAT|A|ATATGACAGCTCAGGTCTTTCCTCTATGCTCAGACTGGTGTGGCTCATCTTCCTTTGCTGCTTTTCATTTTCTGAACT
         >chr3:182355796-182356108 - E=4e-176
GCTGTCACCATTTCTCAGGAACCCTAGAACTGATGCCCTGGGCCTTTTGCCTTTCTTTCATTTCATTCCACCCTTGAGTGAAGGTTTGT

CTGTCTTTGTTTTGTTTGTTTGTTGGGCTTATAATTGTTGACAGAACCCTTGCAAACCTCTCACATAGCTTCTTGGGAAGAGCTGGTTG

GGGCACCACCTACCAGCCTGCACTTCCCTTTCTCAGAGTTTGGAAGCCCCCACCT
```
